# Drug combinatorics and side effect estimation on the signed human drug-target network

**DOI:** 10.1186/s12918-016-0326-8

**Published:** 2016-08-15

**Authors:** Núria Ballber Torres, Claudio Altafini

**Affiliations:** 1 School of Telecommunications Engineering, Universitat Politècnica de Catalunya, 1-3 Jordi Girona Street, Barcelona, 08034 Spain; 2 Division of Automatic Control, Dept. of Electrical Engineering, Linköping University, SE-58183 Linköping, Sweden

## Abstract

**Background:**

The mode of action of a drug on its targets can often be classified as being positive (activator, potentiator, agonist, etc.) or negative (inhibitor, blocker, antagonist, etc.). The signed edges of a drug-target network can be used to investigate the combined mechanisms of action of multiple drugs on the ensemble of common targets.

**Results:**

In this paper it is shown that for the signed human drug-target network the majority of drug pairs tend to have synergistic effects on the common targets, i.e., drug pairs tend to have modes of action with the same sign on most of the shared targets, especially for the principal pharmacological targets of a drug. Methods are proposed to compute this synergism, as well as to estimate the influence of the drugs on the side effect of another drug.

**Conclusions:**

Enriching a drug-target network with information of functional nature like the sign of the interactions allows to explore in a systematic way a series of network properties of key importance in the context of computational drug combinatorics.

**Electronic supplementary material:**

The online version of this article (doi:10.1186/s12918-016-0326-8) contains supplementary material, which is available to authorized users.

## Background

In drug discovery research, moving beyond the “magic bullet” (one-drug, one-target) paradigm is a trend that has become quite popular in recent times, motivated by the discovery of the value of polypharmacology in treating complex diseases [1–3], and by the promises of network pharmacology [4–8], as a “system” way to understand the effects (and side effects) of drugs on an organism. A network viewpoint can help in understanding the effect of a drug on system properties such as robustness, resilience and redundancy, and can be used for system-driven drug discovery [9]. The approach is particularly meaningful to deal with the multifactor nature of many complex diseases, like cancer, asthma, diabetes, neurodegenerative disorders and cardiovascular diseases.

Network representations of drug-target interactions have already been used for several tasks, like to extrapolate information of functional nature on the action of the drugs, to predict novel putative drug-target interactions, and to provide strategies for an efficient use of multidrug therapies, see [10, 11] for an overview. For instance the topological structure and organization of a drug-target network obtained from FDA-approved drugs is investigated in [12], as well as the clustering of its drugs according to the functional categories of the Anatomical Therapeutic Chemical classification. Chemical and genomic information is used in [13] to build a drug-target network for various classes of human targets, like enzymes, ion channels and receptors, using supervised learning. Systematic attempts to identify new drug-target interactions are very frequent in the literature, based on sequence [14], structure [15], pathway-pathway interactions [16], but also on chemical and phenotypical similarity among drugs and sets of ligands [17, 18], often leading to a large number of low-affinity interactions of limited significance. Methods for predicting new potential drug-target interactions for known drugs, using drug-based, target-based and network-based similarity scores are investigated in [19], inspired by algorithms used in the field of recommendation systems, or by random walking the network itself [20]. Drug repositioning is also one of those problems that can be investigated through drug-target networks. Machine learning techniques aimed at extending the target space of already approved drugs are reviewed in [21], while in [22] it is shown how to use constraint based computational methods for metabolic drug repurposing. Another line of research deals with predicting drug combinatorics via gene profiling and gene network reconstruction [23]. As surveyed in [10], the number of datasets and tools specifically dedicated to the analysis of drug-target networks has grown rapidly in the last years.

When investigating a given drug-target network (for instance reconstructed from a database such as DrugBank [24], which is also our database of choice), most computational methods rely principally on the topological information that can be obtained from the bipartite drug-target graph and on the ontology of its constituent components. In this work we aim to add another element of functional nature in the network-based investigation of drug-target interactions, namely the information on the mode of action of the interactions. When browsing DrugBank, many are the possible mechanisms of action of a drug on its targets: it can activate or inhibit the target, it can act as an agonist or an antagonist, as a potentiator or as a blocker, as an inducer or as a suppressor, and so on. Although qualitatively different and applicable to different categories of targets (proteins, macromolecules, nucleic acids, small molecules, etc.), these modes of action can be reasonably classified as *positive* or *negative*. If these and a few more modes of action admit such a signed classification, several more, such as for instance “modulators”, “binder”, “cleavage” (a complete list of categories will be given below) are instead impossible to classify with a sign, and hence cannot be included in the analysis we are proposing in this paper. On the drug-target network, characterizing the modes of action as positive or negative corresponds to associating a sign to the edges of the bipartite graph. Signed graphs have been frequently used in Systems Biology [25–28], and we can draw inspiration from this literature to formulate and solve problems which are meaningful and insightful also for our signed drug-target network.

One such problem, important in the context of multicomponent therapies, is to understand the joint effect of two or more drugs acting on the same targets [29]. To illustrate the point, let us look at two drugs having a common target: if their modes of action have the same sign then it is likely that the combined effect of the two drugs on the target is reinforced. On the contrary, if the modes of action have opposite signs then it is more plausible to assume that the two drugs tend to compensate each other’s action, and hence the overall effect on the target tends to be mitigated. In this paper two actions that have a common sign on a target (regardless of what that sign is) are called *coherent*. They are called *incoherent* when they have opposite signs. Understanding to what extent drug pairs act coherently or incoherently on common targets is an important aspect for instance of drug combinatorics. It is shown in the paper that coherent drug pairs are much more frequent than incoherent, and that the same observation is true when we count drug pairs acting simultaneously on pairs of targets (i.e., we look at length-4 undirected cycles). These basic examples of coherent/incoherent motif counting hint at the signed human drug-target network having a topological and functional (for what can be deduced from the edge signs) organization which is far from random. Such an organization can be investigated in a more systematic way if, rather than common actions on one or two targets, we consider the combined action of drug pairs on the ensemble of common targets. We show in the paper that the distribution of drug pairs acting simultaneously on multiple targets is significantly skewed both towards actions that are coherent on many targets but also towards actions that are incoherent on multiple targets. Neither of these two tails is present in null models. Their meaning is that current drugs tend to interact largely on overlapping targets, often exerting a similar action on all of them (hence leading to coherent drug pairs on multiple targets) but sometimes also exerting antithetical actions on all of them (hence leading to incoherent drug pairs on multiple targets). This is related to the observation that most drugs have a “monochromatic” mode of action (i.e. with same sign) on multiple targets.

By attaching signs to the mechanisms of action, we are also able to quantify the amount of synergism (i.e., when coherence prevails over incoherence) in a drug pair, and to classify all drug pairs accordingly. In the paper we do this systematically for all drug-target interactions that are classified as *pharmacological* in DrugBank, which we take as principal actions (on-target actions) of a drug. The vast majority of drug pairs has a beneficial synergistic action on their common pharmacological drug targets.

Failure of a drug to act properly in clinical trials is often due to unexpected side effects, in the form of drug activity on targets other than the principal targets for which the pharmacological action is intended. Methods to predict in a systematic way such off-target interactions have attracted a considerable attention in recent years [17, 30–33]. In some of these works the side effect is defined phenotypically, in some others based on the off-targets of a drug. If we consider as off-targets of a drug the DrugBank interactions that are not classifiable as pharmacological, then exploiting the signs of the interactions we can try to quantify the action of a drug on the off-targets of a second drug, and in particular seek for drug pairs likely to have a mutualistic benefit on each other’s side effect, for instance drug pairs behaving synergistically on their common principal (pharmacological) targets but having incoherent actions on their common off-targets. An exhaustive classification of all these pairs is carried out for our signed drug-target network.

## Results

Consider the human drug-target network reconstructed from the DrugBank database (see Methods and Table 1), and associate to its edges a sign according to the procedure described in Methods and in Table 2. Table 3 reports the resulting sign distribution. The fraction of edges which cannot be assigned a sign is around 63 %. The fraction decreases drastically if we restrict to actions which can be classified as pharmacological (as defined in the Methods), to less than 11 %.

**Table 1:** Human drug-target network

	Total	Human	Pharmacological actions
n. of drugs	6699	4905	1342
n. of targets	4139	2337	695
total number of edges	16386	11133	2916
n. of signed edges	4624	4128	2599
n. of edges without sign	11762	7005	317

**Table 2:** Drug modes of action and edge signs

Sign	DrugBank actions
Positive	agonist; partial agonist; activator; stimulator; inducer;
	positive allosteric modulator; potentiator; positive modulator
Negative	inhibitor; inhibitory allosteric modulator; inhibitor, competitive; antagonist; partial antagonist; negative modulator; inverse agonist; blocker; suppressor; desensitize the target; neutralizer; reducer
Not classifiable	antibody; cofactor; modulator; binder; chaperone; cleavage; metabolizer; ligand; product of; component of; chelator; cross-linking/alkylation; intercalation; adduct; acetylation; allosteric modulator.

**Table 3:** Signed human drug-target network

	All	Pharmacological actions
n. of drugs	1315	1178
n. of targets	820	578
n. of positive edges	1417	1093
n. of negative edges	2711	1506

**Fig. 1:**
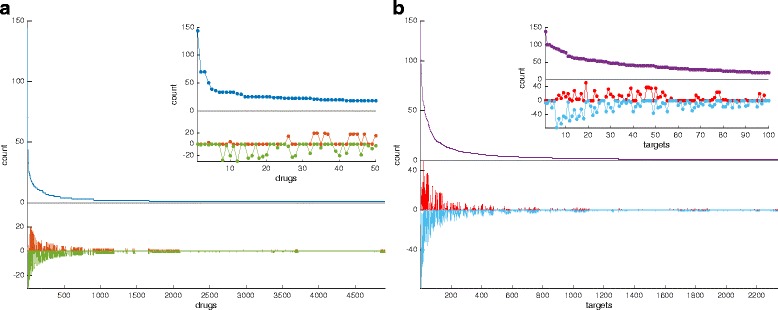
Drug and target connectivity analysis on the human network. Panel **a** The main plot shows the number of human targets associated to each drug. In *blue* the total number of targets is shown, while in red and green are respectively the number of targets for which the mode of action can be classified as positive (*red*) and negative (*green*). The inset shows the same quantities but only for the 50 drugs having the highest total number of targets in DrugBank. Panel **b** The main plot shows the number of drugs associated to each human target. In *violet* the total number of drugs is shown, while in *red* and cyan are respectively the number of drugs with positive (*red*) and negative (*cyan*) modes of action. The inset shows the same quantities but only for the 100 targets having the highest total number of drugs in DrugBank

Looking at the drug degree distribution of Fig. 1a, one can see that the drugs that lack most edge signs are those with higher connectivity, while the situation is much improved for drugs with lower connectivity. Comparing the drug and target connectivity analysis of Fig. 1, the latter has a significant difference in the fact that also targets with high connectivity tend to have a certain fraction (even higher that 50 %) of edges having a known sign. In the drug connectivity histogram, on the other hand, when a drug has signed edges, then they are almost all of the same sign, see inset of Fig. 1a.

Additional file 1: Figure S2 shows the equivalent drug and target connectivity analysis restricted to edges representing pharmacological actions. In both cases the connectivity decreases considerably when we compare it with Fig. 1, and the already mentioned fact that most pharmacological actions have a known sign (see Table 3) allows us to have a sharper picture of the sign distributions. For both drugs and targets, in fact, the sign distribution tends to be “monochromatic”, i.e., most edges adjacent to a drug or to a target tend to be positive or negative but rarely both, see inset plots in Additional file 1: Figure S2.

When restricting the drug-target network to signed edges, we obtain a bipartite signed graph involving 1315 drugs and 820 targets, containing 1417 positive and 2711 negative edges, see Table 3. The drug-target network described in Table 3 has one very large connected component involving 81 % of the drugs and 71 % of the targets, plus a number of other smaller connected components, see Additional file 1: Figure S1.

**Fig. 2:**
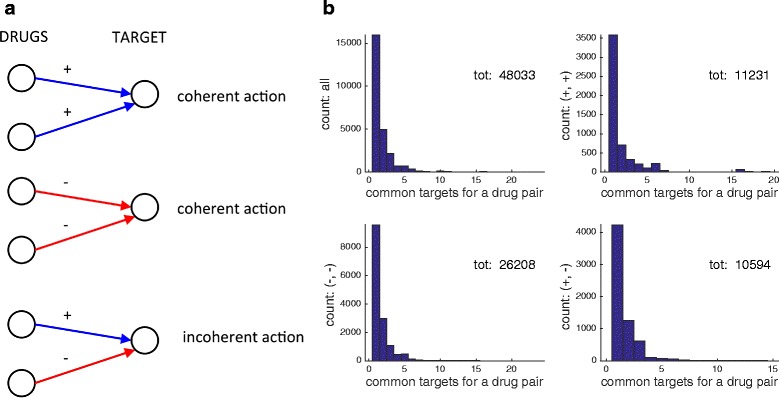
Coherent/incoherent actions of drug pairs. Panel **a** when two drugs act simultaneously on the same target, their action can be coherent (i.e., the second drug tends to reinforce the action of the first) or incoherent (i.e., the second drug tends to counteract the action of the first). Panel **b** drug pairs with common targets, and their sign patterns. All 4 histograms show a count of the number of drug pairs having one or more targets in common (the number of common targets is on the horizontal axis). Only signed edges are considered. *Top left* subpanel: all pairs of drugs having one or more common targets, and considered regardless of sign. *Top right* subpanel: only positive/positive actions. *Bottom left* subpanel: only negative/negative actions. *Bottom right* subpanel: positive/negative actions. The totals refer to the cumulative sums of all pairs

**Combinatorics of signed drugs.** The classification of drug actions into positive and negative modes of action allows us to characterize the effect of multiple drugs acting on the same target. Two drugs sharing the same target tend to reinforce their effect on the target if their modes of action have the same sign, while they tend to mitigate the overall effect if the signs are different. The 3 possible sign combinations of a drug pair on a common target, (+, +), (−, −), and (+, −). are shown in Fig. 2a. The two combinations (+, +) and (−, −) will be referred to as *coherent*, as the action of a drug is reinforced by the presence of a second drug. In the remaining combination (+, −), instead, the presence of a second drug tends to counteract (and hence in general reduce) the action of the first drug. We will call this configuration *incoherent*.

Some statistics on pairs of drugs sharing at least a common target are shown in Fig. 2b. Summing up the data of the top left subpanel of Fig. 2b, the total number of drug pairs incident to at least a common target is 25461. The number of drug pairs having exactly 1 target in common is 15947 (highest bar in top left subpanel of Fig. 2b). The statistics for the three pairs of edge sign combinations (+, +), (−, −) and (+, −) are reported in the remaining 3 subpanels of Fig. 2b. As can be seen in Fig. 2b, incoherent drug actions are not rare in the human drug-target network. The total number of drug pairs acting incoherently on at least one target is 6431. These drug pairs act incoherently on a total of 10594 targets (sum of all bars in the bottom right subpanel of Fig. 2b). The corresponding numbers for coherent actions are however much higher: 20017 drug pairs act coherently on at least one target, for a total number of coherent actions of 37439.

**Fig. 3:**
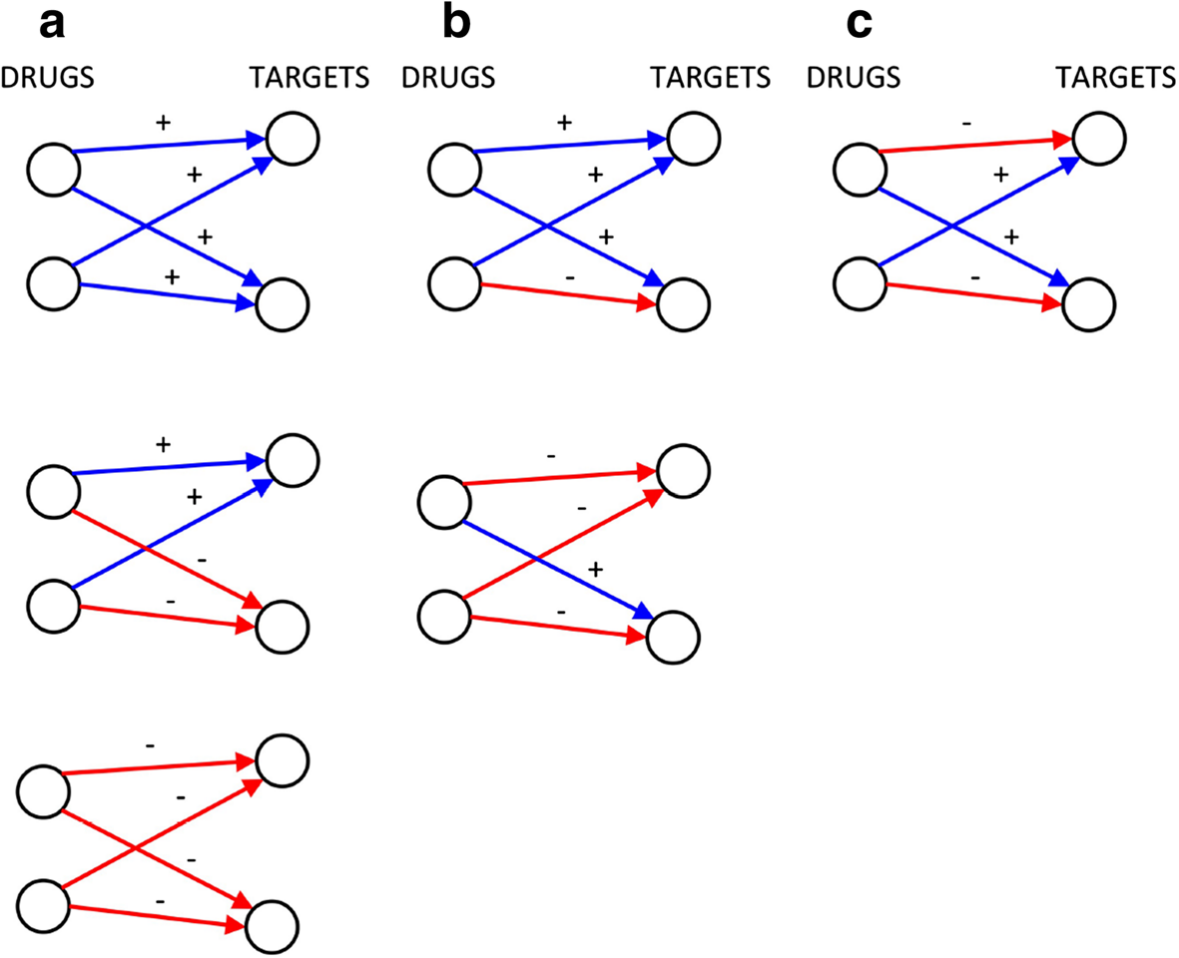
Coherent/mixed/incoherent length-4 cycles. Two drugs having two targets in common in a signed graph. In panel **a** all 3 cases lead to an (undirected) positive cycle, and the drug actions are coherent at each target. In panel **b** instead, the length-4 (undirected) cycle is negative, and the action of the drugs is incoherent on one of the two targets but coherent on the other. In panel **c** the action of the drugs is incoherent on each target, but the cycle is positive

The shortcoming of the analysis carried out so far is that even after restricting to signed actions, 57 % of the 1315 drugs have 2 or more targets, hence when we look at multiple drugs applied simultaneously it is necessary to investigate their effect on *all* of their common targets. As can be seen in Fig. 2b (top left subpanel), around 5000 drug pairs share 2 targets, and more than 2000 have 3 targets in common, while other 2400 pairs have more than 3 targets in common. The simplest possible approach to tackle this more complex problem consists in looking at all cycles of length 4 formed by two drugs having at least 2 targets in common. Cycles are here obviously intended as undirected, i.e., arrows on the edges are dropped. Three qualitatively different classes of length-4 cycles can be identified, which we call (fully) coherent, mixed and (fully) incoherent. They are shown in Fig. 3. Coherent cycles are those for which both drugs act with the same sign on each of the targets, hence reinforcing each other’s action on both targets. In incoherent cycles, instead, the signs of the drug actions are conflicting on both targets, leading to mitigation of the effect on both targets. Mixed cycles occur when the action of a drug pair has the same sign on one of the targets but conflicting signs on the other target (i.e., coherent on one target but incoherent on the other). As can be seen on Table 4, the fraction of coherent length-4 cycles is around 81 *%* of the total, while that of incoherent length-4 cycles is 13.5 %. Mixed cycles are the most rare (around 5.5 %). Notice how fully coherent and fully incoherent length-4 cycles are positive, i.e., they have an even number of negative edges, while mixed cycles are negative (odd number of negative edges). Hence, overall, the fraction of positive length-4 cycles is around 94.5 *%* of the total, see Table 4.

**Table 4:** Coherent and incoherent length-4 cycles

Type of length-4 cycle	n.
coherent, (+, + / +, +)	25158
coherent, (+, + / −, −)	3042
coherent, (−, − / −, −)	28166
mixed, (+, + / +, −)	1138
mixed, (+, − / −, −)	2658
incoherent, (+, − / +, −)	9375

Signs are as in Fig. 3

**Fig. 4:**
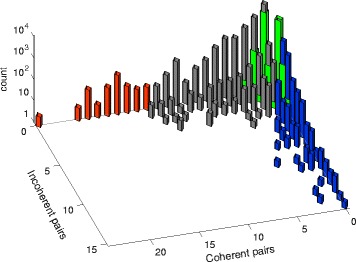
Distribution of coherent/incoherent edge pairs. The red/grey/blue bars of the histogram represent counts of the coherent and incoherent actions of the drug pairs on common targets. The histogram is significantly skewed, meaning that many drug pairs tend to have an abundance of coherent actions or of incoherent actions, rather than of both simultaneously. When the overabundance is statistically significant (binomial cumulative distribution test, p-value of 0.05, see Methods), then the bars are coloured: *red* for overabundance of coherent actions, *blue* for incoherent actions. The green bars overlaying to the other bars represent the distribution of the coherent/incoherent actions for the null model. For nearly all drug pairs, nonzero green bars reach at most 4 targets in common, counting together coherent and incoherent actions, i.e., the two tails are absent in a null model

**Connectivity analysis of drug pairs: distribution of coherent/incoherent actions.** With a total of 48033 targets being acted upon by at least two drugs, a natural question to ask is if such pairwise connectivity is high or low with respect to a null model having the same drug edge distribution. Comparing with a null model, obtained maintaining the same edge distribution (sign included) at the drug side, but reassigning randomly the edges to the targets, then the drug-target topological properties one obtains are drastically different. On a null model, the number of drug pairs sharing at least a target reduces from 25461 to 10126.6 (average over 100 realizations of the null model) and so does the total number of drug pairs incident to a common target, from 48033 to 10388.7. We deduce that the signed drug-target network is highly organized, and in particular highly redundant in its coverage of the targets. For instance, the original target connectivity is such that only near half (416) of the 820 targets are hit by more than one drug, while in a null model this number is around 95 %. However, the target edge distribution for the null model completely lacks the highly connected nodes which can instead be seen in panel (b) of Fig. 1. As can be seen in Fig. 4, the consequence is that in null models a pair of drugs very seldom exceeds a total of 3 common targets, counting both coherent and incoherent actions (green bars). In the true drug-target network, instead, edges tend to concentrate on fewer targets, leading to abundance of targets shared by more than two drugs (in Fig. 4 green bars for the null model should be compare to all the other bars taken together, representing the original network).

Also the sign distribution in the true drug-target network is highly non-random. If 77.9 % of the drug pairs hitting a common target have a coherent action, such number is drastically less in our null models, around 54.8 %, meaning that in the real drug-target network not only known drugs tend to hit always the same targets, but they tend to do so with the same sign, leading to an overabundance of coherent joint actions. In spite of the limited frequency, also the fraction of incoherent drug pair actions is far from being negligible. As can be seen on Fig. 4, the histogram with counts of coherent and incoherent actions of drug pairs is highly skewed and has two tails: just like many drug pairs act coherently on many targets, there exists a considerable number of drug pairs having an incoherent action simultaneously on multiple targets, for instance pairs of drugs in which one of the two activates all its targets while the other inhibit them. The blue bars in Fig. 4 correspond to pairs whose incoherent action is statistically significant, given the total amount of pairs and its coherent/incoherent partition (cumulative binomial test, see “Methods”). Since the number of coherent action pairs is much higher, statistical significance for them requires a pair to hit coherently a larger number of targets (red bars).

**Synergistic/compensatory pharmacological effect of drug pairs.** Most drugs are designed to produce a specific action on selected targets, here denoted *pharmacological targets*. Other targets of the same drug (here called *off-targets*, see Methods) are often present but they are normally undesired. We refer to these off-targets as the *side effect* of the drug.

Let *t*_*i*_ be the number of targets associated to the *i*-th drug for which the sign of the action is available and *p*_*i*_, *p*_*i*_≤*t*_*i*_, the number of signed pharmacological targets of drug *i*. Let further be *c*_*ij*_ the number of common (signed) targets of the drug pair (*i*, *j*), and *p*_*ij*_, *p*_*ij*_≤*c*_*ij*_, the number of (signed) pharmacological targets shared by (*i*, *j*). Denote *s*_*i*_=*t*_*i*_−*p*_*i*_ the number of (signed) off-targets of drug *i* and *s*_*ij*_=*c*_*ij*_−*p*_*ij*_ the number of (signed) off-targets in common between *i*-th and *j*-th drugs. To specify the sign of the action we will use upper indices: $ p_{ij}^{++}$, $ p_{ij}^{--}$ and $ p_{ij}^{+-}$ (respectively $ s_{ij}^{++}$, $ s_{ij}^{--}$ and $ s_{ij}^{+-}$). In this paper we are assuming that the coherent pairs $ p_{ij}^{++} $ and $ p_{ij}^{--} $ enhance the action of each single drug on the common pharmacological targets (i.e., act synergistically), while in an incoherent pair $ p_{ij}^{+-}$ the actions of the two drugs tend to counteract each other (i.e., the action is compensatory). Consider the case *p*_*ij*_>0 and *s*_*ij*_=0, i.e., the two drugs share only common pharmacological targets. Additional file 1: Figure S4 tells us that for pharmacological actions the distribution of coherent/incoherent interaction pairs has a longer tail towards the coherent end, but does not tell us what fraction of pharmacological targets of a drug *i* receives a benefit from the presence of a second drug *j* sharing a certain number of pharmacological targets with it. In order to quantify this, we use the *synergistic score coefficienta*_*i*_(*j*) defined in Methods. This coefficient is by construction between −1 and 1. *a*_*i*_(*j*)>0 means that more coherent than incoherent actions are established on the pharmacological targets of drug *i* by the presence of a second drug *j*. Since *a*_*i*_(*j*)>0 if and only if *a*_*j*_(*i*)>0, this synergistic effect is always mutual, although *a*_*i*_(*j*)≠*a*_*j*_(*i*) when the two drugs have a different number of pharmacological targets. Negative synergistic score means that for a drug pair the incoherent actions are more numerous than the coherent ones. Clearly *a*_*i*_(*j*)<0 if and only if *a*_*j*_(*i*)<0.

**Fig. 5:**
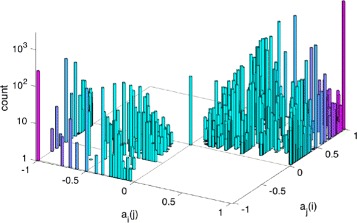
Synergistic scores for drug pairs and pharmacological targets. The histogram shows the distribution of synergistic score coefficients for drug pairs sharing one or more pharmacological targets and no off-targets. When *a*_*i*_(*j*)>0 and *a*_*j*_(*i*)>0, the two drugs induce a benefit on each other’s pharmacological targets, i.e., the number of coherent action pairs is bigger than the number of incoherent ones (81.6 % of the cases)

Of the 14380 drug pairs with common pharmacological targets, 10611 have *p*_*ij*_>0 and *s*_*ij*_=0, hence for them we can compute *a*_*i*_(*j*). The synergistic score coefficient is positive in 81.6 % of the cases. In 45.2 % of the cases it is *a*_*i*_(*j*)≥0.5 and *a*_*j*_(*i*)≥0.5, i.e., a significant mutual synergistic score is produced, see Additional file 2, while in 29.8 % of the cases *a*_*i*_(*j*)≥0.5 but not *a*_*j*_(*i*), see Additional file 3. The 70 cases in which *a*_*i*_(*j*)=*a*_*j*_(*i*)=0 are listed in the Additional file 4, and the 1882 cases in which both *a*_*i*_(*j*) and *a*_*j*_(*i*) are <0 are in the Additional file 5. The histogram of the synergistic score coefficients is shown in Fig. 5.

**Side effect improvement/aggravation through drug combinations.** When two drugs share pharmacological targets but also off-targets, it becomes interesting to understand if it is possible to combine drugs so as to reduce their side effect. The principle that we follow is to look for drug pairs having a positive synergism on common pharmacological targets and incoherent actions on common off-targets. As incoherent actions correspond to opposite edge signs, they tend to compensate each other, hence the side effect tends to be mitigated.

Let us consider drug pairs such that *p*_*ij*_>0 and *s*_*ij*_>0, i.e., having some pharmacological target and some off-target in common. In particular, we look for drug combinations in which

1. the actions on the pharmacological targets are predominantly coherent: $ p_{ij}^{++} + p_{ij}^{--} > p_{ij}^{+-} $;
2. the actions on off-targets tend to cancel each other: $ s_{ij}^{+-}> s_{ij}^{++} +s_{ij}^{--} $.

**Fig. 6:**
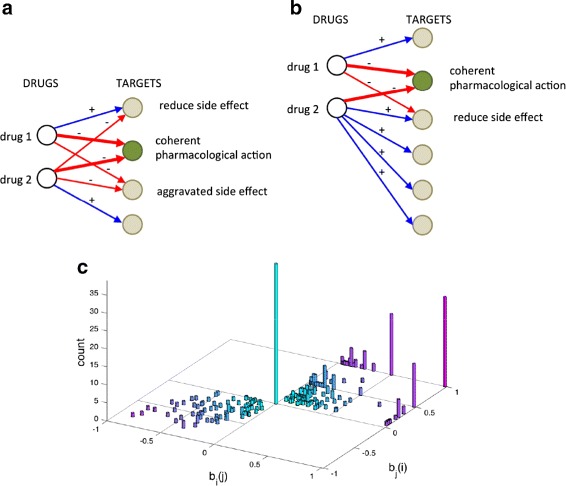
Side effect quantification for drug pairs. Panels **a** and **b** The target in green is a common pharmacological target. The action of the two drugs on it (thick red arrows) is coherent and reinforces the effect. All other targets are off-targets and constitute a side effect for the two drugs. Panel **a** The two drugs share 2 off-targets. In one of them the simultaneous presence of the two drugs tends to mitigate the effect, while in the other the two drugs act with the same sign, hence the side effect on it is aggravated. Overall the drug 1 experiences a side effect improvement on 50 % of its off-targets but an aggravation on the remaining 50 %, while for drug 2 the beneficial/aggravating effect is only on 33 % of the off-targets. For both however, the side effect score is equal to 0: *b*_1_(2)=*b*_2_(1)=0. Panel **b** The side effect on the off-target in common is reduced by the combination of drugs. For drug 1, *b*_1_(2)=0.5, while for drug 2 it is *b*_2_(1)=0.25, because of the higher number of off-targets. Panel **c** The histogram shows the side effect score coefficient for all drug pairs having a positive synergistic score on the common pharmacological targets. For 63 % of the resulting pairs the side effect score is positive, while it is negative for 25 % of pairs and neutral in the remaining cases (bar in the origin, representing cases in which $ s_{ij}^{+-} = s_{ij}^{++} + s_{ij}^{--} $). For a significant fraction of pairs (17 %) the mutual benefit is at least 50 % (i.e., *b*_*i*_(*j*)≥0.5 and *b*_*j*_(*i*)≥0.5). The number increases if we look at pairs in which at least one of the two drugs has a 50 % improvement (42 % of pairs)

To quantify the benefit of adding a second drug *j* on the side effect of drug *i*, we consider the *side effect score coefficientb*_*i*_(*j*) (see Methods). By construction −1≤*b*_*i*_(*j*)≤1, with *b*_*i*_(*j*)<0 meaning an aggravating effect of the drug *j* on the side effect of *i*, *b*_*i*_(*j*)=0 meaning neutral effect in the side effect ($ s_{ij}^{+-} = s_{ij}^{++}+s_{ij}^{--}$, see Fig. 6a), and *b*_*i*_(*j*)>0 meaning an improvement of the side effect of drug *i* due to drug *j*, see Fig. 6b. In particular, *b*_*i*_(*j*)=1 means that the drug *j* hits all off-targets of drug *i* with an action which is opposite in sign to that of drug *i*. Notice that by construction *b*_*i*_(*j*) and *b*_*j*_(*i*) must have the same sign, meaning that for drug pairs with a positive side effect score the benefits are always mutualistic. Screening the 14380 drug pairs having pharmacological targets in common, we obtain 325 pairs. Among these, 205 pairs are beneficial i.e., have *b*_*i*_(*j*)>0 and *b*_*j*_(*i*)>0; 39 are neutral, i.e., have *b*_*i*_(*j*)=*b*_*j*_(*i*)=0 and 81 are aggravating, i.e., *b*_*i*_(*j*)<0 and *b*_*j*_(*i*)<0. The distribution of their side effect score coefficients *b*_*i*_(*j*) is shown in Fig. 6c. For a significant fraction of drug pairs (56 out of 205), both coefficients *b*_*i*_(*j*) and *b*_*j*_(*i*) are ≥0.5, meaning that the drugs *i* and *j* have a significant reciprocal beneficial effect on each other’s side effect. These cases are reported in the Additional file 6. Several more cases (81) are obtained in which *b*_*i*_(*j*)≥0.5 for one of the 2 drugs, although not for both. These cases with “unilateral” side effect improvement are listed in the Additional file 7. They often correspond to cases in which one drug has a large side effect (in terms of number of off-targets) and the other drug a small side effect, see Fig. 6b. Hence when the latter drug is added to the former it can only improve on a few of its many off-targets. The drug pairs giving *b*_*i*_(*j*)=*b*_*j*_(*i*)=0 and those aggravating the side effect are reported in Additional files 8 and 9.

## Discussion

A drug-target network contains valuable information for researchers interested in network pharmacology and drug combinatorics. This has to do not only with the biochemical classification of the drug compounds and with the ontological/therapeutical classification of the molecular targets, but also with the functional classification of the modes of action of the drugs. In order to explore systematically this aspect, we have to introduce a coarse-grained classification of the mechanisms of action. Such a “binary” classification covers a significant fraction of the known categories for drug-target modes of action.

The fact that many human drug-target interactions cannot be classified in terms of action signs is certainly a limitation of the present study and of the approach in general. The fact that the number of such unsigned edges decreases so drastically when we zoom on pharmacological targets (passing from 63 to 11 %) suggests that our results should give reliable predictions on the coherence/incoherence of drug pairs on the “primary” pharmacological targets (and hence on their potential synergies), but could have a limited predictability power on the effective side effect score, when many other unsigned interactions are shared by a pair of drugs alongside the coherent/incoherent off-targets we consider here.

Having so few unsigned pharmacological drug-target interactions also suggests that the modes of action that can be characterized with signs are more “valuable” in terms of describing the therapeutical effects of a drug. Hence also the investigation of the amount of coherence/incoherence encoded in this subset of signed pharmacological interactions is important. An analysis like the one carried out in Figs. 1, 2b and 4 is repeated in the Additional file 1 for the subnetwork of signed pharmacological targets (see Additional file 1: Figures S2, S3 and S4). It turns out that some of the properties mentioned for the entire signed drug-target network still hold for the signed subnetwork of pharmacological actions. For instance, the long tail of coherent action pairs observed in the distribution of Fig. 4 is still visible in Additional file 1: Figure S4, much more than the corresponding incoherent tail, in accordance with the high synergistic score we found.

Moving beyond drug pairs (to triplets, quadruplets, etc.) is a more challenging problem. A possible approach consists in computing the so-called level of structural balance [34], i.e., the amount of “disorder” that characterizes the drug-target network as a whole, intending with that the amount of contradictory “orders” that multiple drugs send to their common targets [25, 26]. Such information, which is particularly important in a network perspective, is carried out in the Additional file 1. Its limitation is that it does not allow to distinguish between coherent and incoherent edge pairs, but only between positive (undirected) cycles and negative (undirected) cycles. Only the latter contribute to the disorder of the network.

When classifying the common targets of drug pairs according to principal targets and off-targets, it can be observed that in the vast majority of cases in which drug pairs share common pharmacological targets they have no common off-target, meaning that the synergistic score coefficient describes the entire overlap of the pair. The number of drug pairs with significantly large synergistic score is fairly big (4799 in Additional file 2 and 3163 on Additional file 3). For many of these pairs sharing one or more common pharmacological targets, experimental evidence of synergistic action is available. For instance the DNA antimetabolites Gemcitabine and Fludarabine share an inhibitory action on ribonuceotide reductase, hence we classify them as synergistic. They are known to act synergistically as anticancer agents in Acute Myeloid Leukemia [35]. For other pairs, there is no documented improvement by the simultaneous application of the two drugs. For instance, Panitumumab and Cetuximab are both monoclonal antibodies targeting the epidermal growth factor receptor, and used for treating EGFR-expressing metastatic colorectal cancers. Although they are said to have similar activity [36], they are also though to differ in their isotype and possibly in their mechanisms of action (Panitumumab is known to be inefficient for certain type of mutations [37]). In our metric the synergistic scores of the pair are both 1, just based on the common target. In other cases the improvement, as measured by the synergistic score, is positive but low because of the limited overlap among the pharmacological targets. One example among many is the pair Propofol and Sevoflurane. Both have the GABA_A_ receptor among their pharmacological targets. It is known [29] that the mechanism of action is “positive allosteric modulation” for both (here “ + ”), although their actions occur on different sites of the same target. In fact, their joint action is classified as additive in [29]. Given that Propofol has two more pharmacological targets and Sevoflurane has five more (plus, for both, several off-targets, none in common), their *a*_*i*_(*j*) coefficients are positive but low.

In oder to make a more systematic validation of the synergistic/compensatory effects described in this paper, we downloaded the DCDB database [38] (http://www.cls.zju.edu.cn/dcdb/), containing ∼1360 experimental combinations of two or more drugs. Around ∼1000 of these combinations are for human targets, and of these 118 overlap with the drug pairs sharing at least one target considered in this study (in the remaining experiments the drugs do not hit the same target, hence do not overlap with our subset of drug pairs). The drug combinations of DCDB are labeled as “efficacious” if experimental evidence is available of improved benefits (80 out of 118) “non-efficacious” for unsuccessful usages (28 out of 118), and “need further study” when unclear (10 out of 118). The majority of these 118 drug combinations are classified as (+, +) actions (31 cases) or (−, −) actions (72 cases). The vast majority of drug pairs sharing a common target for which experimental evidence is available confirms the synergistic effect we are suggesting in this paper. In particular this is true for the (+, +) pairs (24 out of 31), less for the (−, −) pairs (44 out of 72). The complete list is given in Additional file 10.

For what concerns the drug pairs influencing each other’s side effect, their number is remarkably low. This is probably due to the fact that often times when *s*_*ij*_>0 the synergistic score *a*_*i*_(*j*) is negative, i.e., the pair has a majority of compensatory effects on its common pharmacological targets. These pairs are not considered in our analysis (they belong to the incoherent tail of the histogram in Fig. 4). Nonetheless, if we look at pairs with positive synergistic score and acting nontrivially on each other’s side effect, a few interesting categories emerge. For what concerns the drug pairs having a high reciprocal benefit on the side effect, many of the cases listed in Additional file 6 deal with opioid receptors, where analgesics with agonist/antagonist action on different receptors are well-known. In particular drug pairs having a *μ*-type opioid receptor as coherent pharmacological target and some other opioid receptor (like *δ*-type or *κ*-type) as incoherent off-targets are quite frequent, see Additional file 6. Many different drug pairs (legal or less) show this kind of mutualistic benefit on the majority of their off-targets. Another family of examples found in Additional file 6, concerns drugs acting as agonists or partial agonists on *β*_1_ adrenergic receptors, but having opposite effects on *β*_2_ adrenergic receptors (here considered as off-target of several drugs, according to DrugBank pharmacological action classification). An example of a pair of drugs having *b*_*i*_(*j*)=*b*_*j*_(*i*)=1 is given by Modafinil and Lisdexamfetamine. Both act as inhibitors on the sodium-dependent dopamine transporter, but the first drug is a partial agonist for the Alpha-1B adrenergic receptor, while the second is an antagonist for the same target, hence these actions tend to cancel each other. Both drugs have no other known target. As an example of a pair for which *b*_*i*_(*j*)=1 and *b*_*j*_(*i*) positive but small (Additional file 7), we can mention Icosapent and Rosiglitazone. Both have an agonist action on Peroxisome proliferator-activated receptor *γ* (pharmacological target), while they have incoherent actions on the off-target Long-chain-fatty-acid–CoA ligase 4 (one is an inducer, while the other is an inhibitor). While Rosiglitazone has no other target, Icosapent has 8 more targets, hence the two side effect score coefficients are 1 and 1/9. A pair for which the side effect worsen is given by Amitriptyline and Paliperidone (see Additional file 9). Both have a large number of targets (33 for Amitriptyline and 17 for Paliperidone), only a fraction of which are pharmacological (1 for Amitriptyline and 5 for Paliperidone). Both act as antagonist on the common pharmacological target 5-hydroxytryptamine receptor 2A, but they also share 5 off-targets, on 4 of which (mostly adrenergic receptors) the action is coherent. Only on the Histamine H1 receptor they exert an incoherent action (one as antagonist, the other as agonist), hence overall both *b*_*i*_(*j*) and *b*_*j*_(*i*) are negative.

In DrugBank a set of “BioInteractions” (i.e., drug-drug interactions) is reported for each drug, based on common targets, but also on other mechanisms like assimilation and clearance (using information on enzymes and transporters affecting the drugs). These drug-drug interactions are limited to pharmacological targets, and essentially overlap with the pharmacological drug pairs used in our analysis and shown for instance in Additional file 1: Figure S4. Having lumped together many modes of action into positive and negative signs, however, allows us to perform a further step, namely to quantify the level of synergism of these drug-drug interactions and hence search for pairs with high synergistic benefit. Furthermore, DrugBank biointeractions do not take into account off-targets as we do here, hence no investigation of side effect is possible at all if we limit ourself to the information currently provided by DrugBank.

It is worth observing that the presence of positive and negative signs on the interactions and the limited knowledge of the drug-target dose-response curves (for single drugs and for drug pairs) mean that classical definitions of synergism, as given in the framework of epistasis analysis or in drug combination theory [39], cannot be applied in our context. The methodology adopted in this paper, counting the overlap of two drugs and splitting it into coherent and incoherent, seems to us the simplest possible way to generalize the notion of synergism to the present context.

Clearly, as also some of the examples mentioned above show, our definitions of synergism and side effect can be oversimplified in certain cases. More sophisticated variants of the approach discussed here consist for instance in replacing the main target/off-target distinction that we use with some other criteria, like an ontological classification of the main targets of a drug (often known), or a colocalization on a specific pathway of interest for a disease. For instance many cases of synergistic therapeutic reinforcements happen because different drugs act on different targets, located for example on redundant branches of the same pathway or on distinct but complementary pathways [29]. As a rule of thumb, when the synergism is due to indirect mechanisms, it is more difficult to capture in large-scale models. A classical example is augmentin, a combination of amoxicillin and clavulanic acid [7]. The calvulanic acid inhibits one of the degradation pathways of amoxicillin hence exerting an indirect, yet strong, synergistic effect. Another related shortcoming of the analysis performed here is that drugs are considered as on/off. Clearly dosage and timing of compound application are factors that could be taken into account in a model. For instance, the idea that two drugs having modes of action with the same sign lead to an enhanced effect on a common target is more likely to happen at low dosage than at saturation, where only the strongest drug is likely to bind to the target (and competitive, instead of cooperative, effects may appear). In some cases, the information on pharmacodynamics, assimilation and clearance mechanisms available in DrugBank could be used for this scope, but it is unlikely that a systematic analysis can be built on these bases and be feasible at network level. The caveat in this case is that very little can be said on how data on single drug response remain valid for mixing of drugs, unless extensive experimental evaluation is performed. The broad range of possibilities involving drug pairs, listed for instance in [29, 39] clearly shows that only direct experimental evidence can provide an irrefutable classification of combined modes of action. Nevertheless, we believe that our sign-based classification can provide a number of clues to understand drug-target networks at functional level, and help shed light into its (network-wide) functional organization.

## Conclusions

In this paper we have introduced the idea that the mode of action of a drug-target interaction can be used to construct a signed drug-target network. Characterizing the edges of a drug-target network with sign adds a connotation of functional nature to the network, and enables the (network-wide) investigation of a series of properties of importance in the context of drug combinatorics.

## Methods

A drug-target network is constructed using the *DrugBank* dataset (version 4.3, downloaded November 2015). It is a bipartite graph having two classes of nodes: drugs and targets. The edges represent known actions of a drug on a target. Both FDA approved and experimental drugs are considered. The data for this network are given in Table 1. About 68 % of the total of DrugBank drug-target interactions are associated to human targets, see again Table 1. Selecting only human targets, a subnetwork can be extracted. It is on this subnetwork that the paper is focused.

**Pharmacological actions and off-targets.** The drug-target actions (i.e., edges of the drug-target graph) can be subdivided into “pharmacological actions” and “unknown” or “no pharmacological action”. Normally the pharmacological actions correspond to “main targets”, for which the drug has been designed and the remaining to “off-target actions”, i.e., side effects of the drug, see Table 1 and Additional file 11.

**Sign of the drug-target actions.** The vast majority of categories used by DrugBank to describe how a drug acts on a target can be classified into 2 modes of action: “positive” or “negative”. The specific categories falling into the two modes are listed in Table 2. Some categories, like “antibody”, “cofactor”, etc. cannot be classified as positive or negative edges, and hence are not associated to any sign, see again Table 2. See also the adjacency matrix in Additional file 11.

**Skewness of the coherent/incoherent drug pairs distribution.** Consider the signed human drug-target network. For the drug pair (*i*, *j*), let *c*_*ij*_ be the number of targets in common, split into $ c_{ij}^{++},$$ c_{ij}^{--} $ and $ c_{ij}^{+-}$ according to the signs of the corresponding drug-target edges. Denote $ \xi = c_{ij}^{++} + c_{ij}^{--} $ the total number of coherent edge pairs for the drug pair, and

$$\rho= \frac{\sum_{i,j} \left(c_{ij}^{++} + c_{ij}^{--}\right) }{\sum_{i,j} c_{ij} } $$

the probability of an edge pair to be coherent over the entire network. Then the probability of drawing at most *ξ* coherent edge pairs out of *c*_*ij*_ edge pairs is the cumulative binomial

1 $$ P[ x \leq \xi]= \sum_{\ell =0}^{\xi} \left(\begin{array}{c} c_{ij} \\ \ell \\ \end{array} \right) (1-\rho)^{\ell} \rho^{c_{ij}-\ell}.   $$

If *α*=0.05 is the threshold for statistical significance, when the *p*-value 1−*P*[*x*≤*ξ*]<*α* the drug pair (*i*, *j*) is enriched for coherent actions. A similar calculation applies also for the significance of the incoherent actions of the pair (*i*, *j*).

**Quantification of pharmacological synergism in drug pairs.** If *p*_*ij*_ is the number of signed pharmacological targets shared by the drugs *i* and *j* (split, according to the signs, into $ p_{ij}^{++}$, $ p_{ij}^{--}$ and $ p_{ij}^{+-}$, such that $ p_{ij}^{++}+ p_{ij}^{--}+ p_{ij}^{+-} = p_{ij}$), then the *synergistic score coefficient* is defined as

$$a_{i}(j) = \frac{p_{ij}^{+-}-(p_{ij}^{++} +p_{ij}^{--})}{p_{i}}. $$

Since *p*_*ij*_≤*p*_*i*_, it must be *a*_*i*_(*j*)∈[−1, 1], and, by construction, sign(*a*_*i*_(*j*))=sign(*a*_*j*_(*i*)). Targets that are classified as pharmacological for one drug of a pair but not for the other are not counted in *p*_*ij*_.

**Side effect quantification in drug pairs.** Let *s*_*i*_ be the side effect of the *i*-th drug, i.e., the number of human targets that are not classified in DrugBank as pharmacological targets. If *s*_*ij*_ is the number of common targets of the drug pair (*i*, *j*) which are not pharmacological targets for at least one of the two drugs, and $ s_{ij}^{++}$, $ s_{ij}^{--}$ and $ s_{ij}^{+-}$ specify theirs signs, then the *side effect score coefficient* is defined as

$$b_{i}(j) = \frac{s_{ij}^{+-}-\left(s_{ij}^{++} +s_{ij}^{--} \right)}{s_{i}}. $$

Clearly *b*_*i*_(*j*)∈[−1, 1], and, by construction, sign(*b*_*i*_(*j*))=sign(*b*_*j*_(*i*)).

## Abbreviations

FDA, Federal Drug Administration

## Additional files

Additional file 1 Supplementary text and figures (pdf file). (PDF 125 kb)

Additional file 2 Text file containing all drug pairs having synergistic scores *a*_*i*_(*j*)≥0.5 and *a*_*j*_(*i*)≥0.5. (TXT 1903 kb)

Additional file 3 Text file containing all drug pairs having synergistic scores *a*_*i*_(*j*)≥0.5 but 0<*a*_*j*_(*i*)<0.5. (TXT 2664 kb)

Additional file 4 Text file containing all drug pairs having synergistic scores *a*_*i*_(*j*)=*a*_*j*_(*i*)=0. (TXT 40 kb)

Additional file 5 Text file containing all drug pairs having negative synergistic scores *a*_*i*_(*j*)<0 and *a*_*j*_(*i*)<0. (TXT 1082 kb)

Additional file 6 Text file containing all drug pairs having side effect scores *b*_*i*_(*j*)≥0.5 and *b*_*j*_(*i*)≥0.5. (TXT 19 kb)

Additional file 7 Text file containing all drug pairs having side effect scores *b*_*i*_(*j*)≥0.5 but 0<*b*_*j*_(*i*)<0.5. (TXT 76 kb)

Additional file 8 Text file containing all drug pairs having side effect scores *b*_*i*_(*j*)=*b*_*j*_(*i*)=0. (TXT 44 kb)

Additional file 9 Text file containing all drug pairs having negative side effect scores *b*_*i*_(*j*)<0 and *b*_*j*_(*i*)<0. (TXT 113 kb)

Additional file 10 Drugbank_Overlap_drugpairs_with_DCDB. Text file containing all drug pairs overlapping with the experimental drug combination of the database DCDB. (TXT 8 kb)

Additional file 11 Drug_Target_Signed_Network.mat. Matlab file containing the drug-target adjacency matrix. (TXT 28 kb)
