# Supplementary material for: Drug combinatorics and side effect estimation on the signed human drug-target network
Source: BMC Syst Biol. 2016 Aug 15;10:74. doi: 10.1186/s12918-016-0326-8 (PMC4986181; doi:10.1186/s12918-016-0326-8)
Supplement: Additional file 1 — Supplementary text and figures (pdf file). (PDF 125 kb) [file 12918_2016_326_MOESM1_ESM.pdf]

SUPPLEMENTARY MATERIAL
Drug combinatorics and side effect estimation on the signed human drug-target network

Núria Ballber Torres[1] and Claudio Altafini[2*]
[1] School of Telecommunications Engineering, Universitat Politècnica de Catalunya
1-3 Jordi Girona Street, 08034 Barcelona, Spain.
[2] Division of Automatic Control, Dept. of Electrical Engineering,
Linköping University, SE-58183, Linköping, Sweden.

March 31, 2016

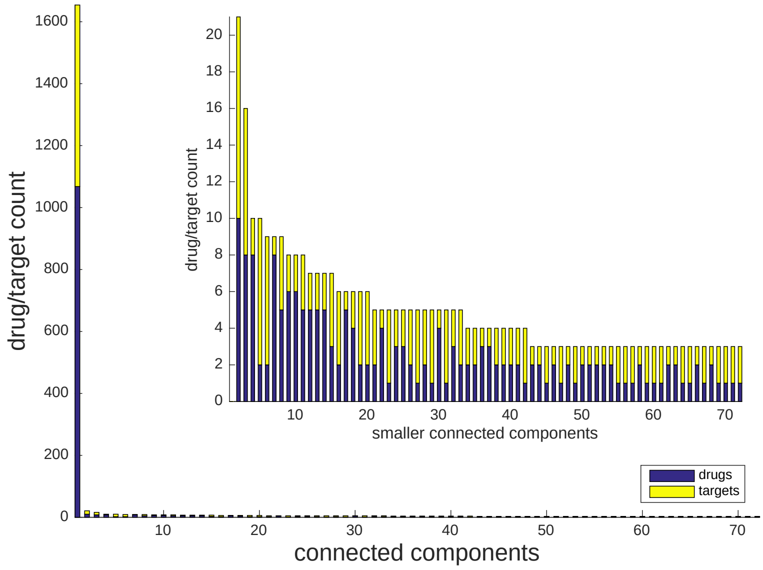

Figure S1: **Connected components of the signed drug-target network**. The main plot shows that the network has a very large connected component (of size 1654, of which 1068 drugs and 586 targets out of a total of 1315+820=2135 nodes). It has then other 133 smaller connected components, whose number of drug/targets can be seen in the inset plot (at least for those of dimension larger than 2).

Combinatorial analysis restricted to pharmacological drug pairs. The analysis carried out in the paper for all signed drug-target interactions is here repeated restricting to the signed pharmacological drug-target pairs (see Tables 1 and 3 of the paper for a quantification). In particular the equivalent of Figs. 1, 2(b) and 4 of the paper is shown in Figs. S2, S3 and S4. As already commented in the paper, a sign can be associated to most pharmacological drug-target

*Corresponding author: claudio.altafini@liu.se

interactions, which leads to a more clear picture of the connectivity properties on both the drug side and the target side, see Fig. S2. Notably, most drugs have outgoing edges with the same sign, and most targets have incoming edges with the same sign. As a consequence, the number of coherent actions of drug pairs on common targets increases slightly, and passes from 77.9% of all pairs to 84.3% (compare Fig. 2(b) and Fig. S3). However, as Fig. S4 shows, the doubly skewed profile of Fig. 4 is still observable, although the tail of coherent pairs is now much longer than the one of incoherent pairs.

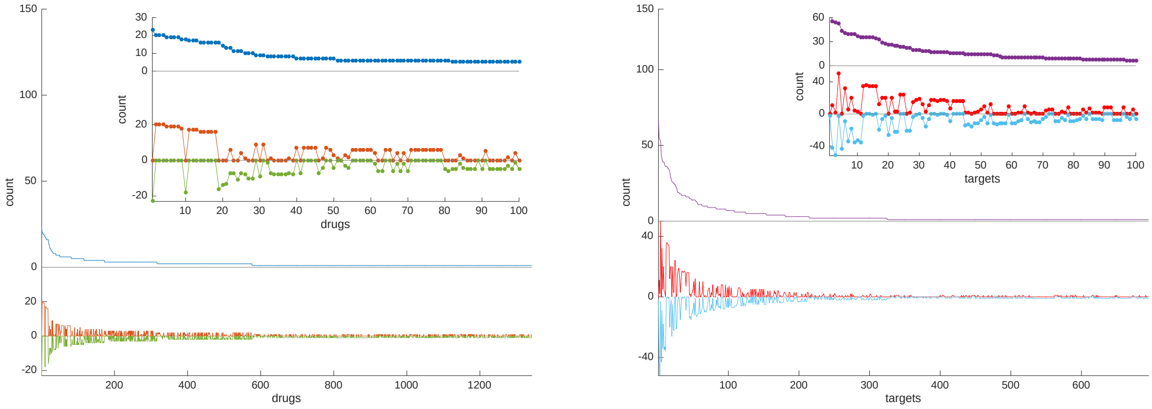

A B

Figure S2: **Drug and target connectivity analysis on the human network (pharmacological actions).** The same quantities as in Fig. 1 of the paper are shown, limited to the edges that have a known pharmacological action. The number of targets per drugs (panel (a)) is now smaller, and almost all of them have a sign. Notice how the action of a drug on its targets is often only positive or only negative. The target connectivity (panel (b)) is higher than the drug connectivity. Often, but not always, there is a neat majority of drugs acting with the same sign.

Higher order combinatorics of drugs: estimating the level of structural balance. Characterizing in a synthetic way what happens when more than 2 drugs are applied simultaneously is a difficult problem because of combinatorial explosion. One possibility consists in putting together all cycles (of any length) which are positive, regardless of their coherence/incoherence, and estimating the so-called *level of structural balance* of the overall drug-target signed graph [1, 2]. The rationale behind this approach lies in the observation that if a graph has only positive cycles (directed and undirected, see [4]), the corresponding dynamical system will behave as a monotone system, i.e., a system having an ordered and predictable response to perturbations (here drug responses), with no oscillations and no chaotic behavior [4]. For signed graphs, an alternative, yet equivalent, concept associated with the sign of the cycles is that of *disorder*, inspired by the Statistical Physics notion of frustration in an Ising spin glass [3], see [1] for a full explanation. Also in this case it can be intended as a way to capture the amount of contradictory orders that multiple drugs send to their common targets. Restricting to length-4 cycles, the mixed cycles of Fig. 3(b) of the paper correspond to contradictory orders that the two drugs send to the two targets (for target 1 both drugs have the same effect, while for target 2 they have opposite effects). Counting the signs of the edges, the cycle is negative. The incoherent length-4 cycle of Fig. 3(c) is instead positive, and in fact the pair of signs of the actions of the two drugs is the same on each of the two targets (incoherent on both). When a signed graph has negative cycles then it cannot be exactly monotone (i.e., it has some disorder).

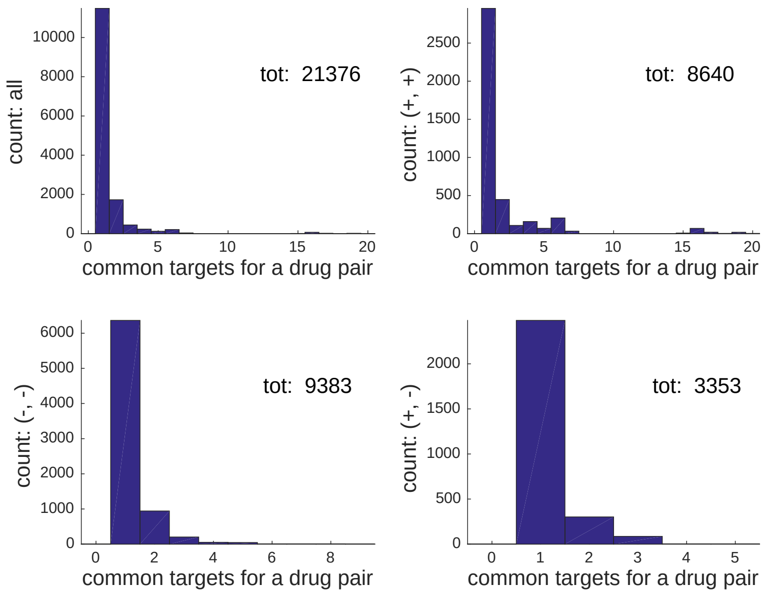

Figure S3: **Drug pairs with common targets, and their sign patterns (pharmacological actions)**. Analogous of Fig. 2(b) of the paper, but restricted to signed pharmacological interactions. All 4 histograms show a count of the number of drug pairs having one or more pharmacological targets in common (the number of common pharmacological targets is on the horizontal axis). Top left panel: all pairs of drugs having one or more common pharmacological targets, and considered regardless of sign. Top right panel: only positive/positive pharmacological actions. Bottom left: only negative/negative pharmacological actions. Bottom right: positive/negative pharmacological actions. The totals refer to the cumulative sums of all pairs.

However, when the fraction of negative cycles is small, then it will be near-monotone (i.e., it will have a small amount of disorder). Several biological networks have been shown to behave in this way, see [2]. The high percentage of positive length-4 cycles present in our drug-target network strongly suggests that it must be near-monotone. Estimating how close to monotone a signed graph is requires to compute the minimal number of edges whose sign must be changed in order to obtain a graph with only positive cycles. Such a number is referred to as *level of structural balance*. (It is sometimes called the “distance to monotonicity” [2] or the “amount of frustration” in Statistical Physics, in reference to the computation of the ground state of an Ising spin glass, see [3]). Although computing the exact level of structural balance is a hard problem, several efficient heuristics are available for networks of the size of our drug-target network. When we apply the heuristic described in [1] to the signed human drug-target network, we obtain that the best estimate of the level of structural balance is 243, i.e., the network is 243 edges “away” from having all positive cycles (of any length). This is one order of magnitude less than the number of mixed length-4 cycles described in Table 4, meaning that those cycles are not independent but largely come from the same edges. That such a number is indeed very low (and hence that our drug-target network behaves near-monotonically) is confirmed if we compare the level of structural balance induced by the true sign pattern with that induced by null models having exactly the same topology and the same number of positive/negative edges as our drug-target network but in which the signs have been redistributed at random on the graph. Fig. S5 shows that the best estimate of the level of structural balance in 300 realizations of the null model (green bars) is always much higher than the best estimate on the true model (red bar). A factor of at least 6 separates the null models from the true edge sign distribution,

confirming that the true sign distribution of the drug-target actions is far from random. It is actually organized so that most drugs act in a similar way on most of their targets. This result is coherent with the monochromatic edge sign distribution visible in Fig. 1, especially in the drugs connectivity pattern.

Unfortunately the procedure does not allow to distinguish between coherent and incoherent positive cycles (and further sub-classes that appears when the cycles have length higher than 4).

References

[1] G. Facchetti, G. Iacono, and C. Altafini. Computing global structural balance in large-scale signed social networks. *Proc. Nat. Ac. Sci.*, 108:20953–8, 2011.

[2] Giovanni Iacono, Fahimeh Ramezani, Nicola Soranzo, and Claudio Altafini. Determining the distance to monotonicity of a biological network: a graph-theoretical approach. *IET Syst. Biol.*, 4(3):223–235, 2010.

[3] M. Mezard, G. Parisi, and M. Virasoro. *Spin glass theory and beyond*. World Scientific, 1986.

[4] Eduardo D. Sontag. Monotone and near-monotone biochemical networks. *Syst. Synth. Biol.*, 1(2):59–87, 2007.

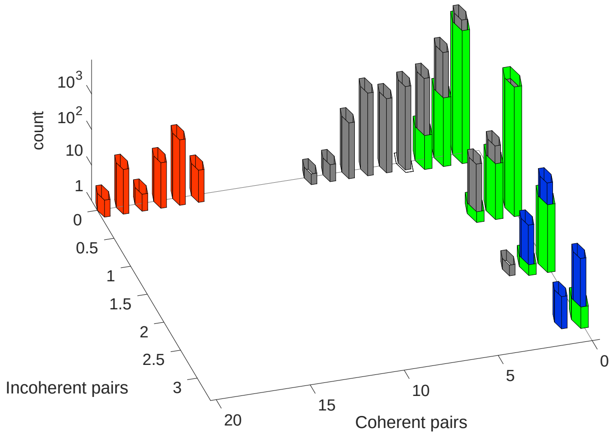

Figure S4: **Coherent/incoherent edge pairs distribution (pharmacological actions).** The red/grey/blue bars of the histogram represent counts of the coherent and incoherent actions of the drug pairs on common pharmacological targets. The histogram is significantly skewed, meaning that many drug pairs tend to have an abundance of coherent actions or of incoherent action rather than of both simultaneously. When the overabundance is statistically significant (binomial cumulative distribution test, p-val of 0.05, see Materials and Methods), then the pairs are coloured: red for overabundance of coherent actions, blue for incoherent actions. Notice the presence of a long tail towards the coherent actions (red bars). The green bars overlaying to the other bars represent the distribution of the coherent/incoherent actions for the null model. For nearly all drug pairs, nonzero green bars reach at most 4 targets in common, counting together coherent and incoherent pharmacological actions, i.e., the tail of coherent pairs is absent in a null model.

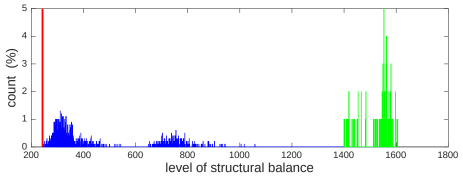

Figure S5: **Level of structural balance.** The histogram shows in blue the result of 1000 runs of our algorithm for estimation of the level of structural balance of the true drug-target network. The lowest of these values (243) is the best estimate we obtained (red bar). In green the estimation of the level of structural balance on 300 null models having the same topology of our drug-target network and the same amount of positive and negative edges, but with positions reshuffled. For each reshuffling, 1000 runs of the same algorithm are performed, and only the best estimate of each reshuffling is shown.
